# Association between sodium-glucose cotransporter-2 inhibitors and incident atrial fibrillation/atrial flutter in heart failure patients with reduced ejection fraction: a meta-analysis of randomized controlled trials

**DOI:** 10.1007/s10741-022-10281-3

**Published:** 2022-10-25

**Authors:** Dimitrios Sfairopoulos, Tong Liu, Nan Zhang, Gary Tse, George Bazoukis, Konstantinos Letsas, Christos Goudis, Haralampos Milionis, Apostolos Vrettos, Panagiotis Korantzopoulos

**Affiliations:** 1 First Department of Cardiology, University of Ioannina Medical School, Ioannina, Greece; 2 Tianjin Key Laboratory of Ionic-Molecular Function of Cardiovascular Disease, Department of Cardiology, Tianjin Institute of Cardiology, Second Hospital of Tianjin Medical University, Tianjin, China; 3 Kent and Medway Medical School, Kent, UK; 4 Department of Cardiology, Larnaca General Hospital, Larnaca, Cyprus; 5 Laboratory of Cardiac Electrophysiology, Onassis Cardiac Surgery Center, Athens, Greece; 6 Department of Cardiology, Serres General Hospital, 45110 Serres, Greece; 7 Department of Internal Medicine, Faculty of Medicine, School of Health Sciences, University of Ioannina, Ioannina, Greece; 8 Department of Cardiology, Royal Brompton and Harefield Hospitals, London, UK

**Keywords:** SGLT2 inhibitors, Heart failure, Atrial fibrillation, Atrial flutter

## Abstract

Atrial fibrillation (AF) and atrial flutter (AFL) are associated with adverse outcomes in patients with heart failure and reduced ejection fraction (HFrEF). We investigated the effects of sodium-glucose cotransporter-2 inhibitors (SGLT2i) on the incidence of AF and/or AFL in HFrEF patients. PubMed and ClinicalTrials.gov were systematically searched until March 2022 for randomized controlled trials (RCTs) that enrolled patients with HFrEF. A total of six RCTs with 9467 patients were included (*N* = 4731 in the SGLT2i arms; *N* = 4736 in the placebo arms). Compared to placebo, SGLT2i treatment was associated with a significant reduction in the risk of AF [relative risk (RR) 0.62, 95% confidence interval CI 0.44–0.86; *P* = 0.005] and AF/AFL (RR 0.64, 95% CI 0.47–0.87; *P* = 0.004). Subgroup analysis showed that empagliflozin use resulted in a significant reduction in the risk of AF (RR 0.55, 95% CI 0.34–0.89; *P* = 0.01) and AF/AFL (RR 0.50, 95% CI 0.32–0.77; *P* = 0.002). By contrast, dapagliflozin use was not associated with a significant reduction in the risk of AF (RR 0.69, 95% CI 0.43–1.11; *P* = 0.12) or AF/AFL (RR 0.82, 95% CI 0.53–1.27; *P* = 0.38). Additionally, a “shorter” duration (< 1.5 years) of treatment with SGLT2i remained associated with a reduction in the risk of AF (< 1.5 years; RR 0.58, 95% CI 0.36–0.91; *P* = 0.02) and AF/AFL (< 1.5 years; RR 0.52, 95% CI 0.34–0.80; *P* = 0.003). In conclusion, SGLT2i therapy was associated with a significant reduction in the risk of AF and AF/AFL in patients with HFrEF. These results reinforce the value of using SGLT2i in this setting.

**Supplementary Information:**

The online version contains supplementary material available at 10.1007/s10741-022-10281-3.

## Introduction

Atrial fibrillation (AF) and heart failure with reduced ejection fraction (HFrEF) frequently coexist, and each affects the course and treatment of the other [1]. In patients with HFrEF, AF, especially if new-onset or paroxysmal, has been associated with a significantly increased risk of adverse outcomes [2]. Additionally, atrial flutter (AFL), although much less well studied, is considered to have a similar clinical impact [3]. Furthermore, AF and AFL impose significant therapeutic challenges in the setting of HFrEF [4]. Therefore, prevention of these arrhythmias represents an important target in patients with HFrEF and treatments that reduce their burden may yield considerable clinical benefits.

Sodium-glucose cotransporter-2 inhibitors (SGLT2i) have been shown to reduce the risk of HF hospitalization and cardiovascular mortality in patients with HFrEF and represent an effective novel therapeutic modality for this condition [4–8]. Interestingly, recent clinical evidence is also indicative of a potential therapeutic effect of SGLT2i on AF and/or AFL. In particular, a post hoc analysis of the Dapagliflozin Effect on Cardiovascular Events-Thrombolysis in Myocardial Infarction 58 (DECLARE-TIMI 58) trial demonstrated that dapagliflozin decreases the relative risk of AF/AFL in individuals with type 2 diabetes and high cardiovascular risk [9]. Furthermore, recent meta-analyses have shown that treatment with SGLT2i was associated with a lower incidence of AF and AF/AFL in individuals with type 2 diabetes [10], as well as in mixed populations comprised of patients with type 2 diabetes, chronic kidney disease, and HF [11–15]. However, to the best of our knowledge, no randomized controlled trials (RCTs) or meta-analyses have addressed the relationship between SGLT2i and these arrhythmias in patients with HFrEF.

Therefore, a systematic review and meta-analysis of RCTs that enrolled patients with HFrEF was performed, examining serious adverse event reports of AF and/or AFL according to randomized assignment to SGLT2i or placebo. Furthermore, subgroup analyses were conducted to compare the SGLT2i treatment effect between different drug types and length of follow-up.

## Methods

This meta-analysis was conducted according to the Cochrane Handbook (Version 6.3) and the Preferred Reporting Items for Systematic Reviews and Meta-Analyses (PRISMA) guidelines.

### Search strategy

PubMed and ClinicalTrials.gov were systematically searched for eligible studies from their inception to March 03, 2022. We screened published and unpublished RCTs using the following search strategy: (sodium-glucose cotransporter-2 inhibitors OR SGLT2 inhibitors OR SGLT-2 inhibitors OR SGLT 2 inhibitors OR empagliflozin OR dapagliflozin OR canagliflozin OR ertugliflozin OR tofogliflozin OR ipragliflozin OR luseogliflozin OR remogliflozin OR sergliflozin OR sotagliflozin) AND (heart failure OR heart failure with reduced ejection fraction OR HFrEF OR atrial fibrillation OR atrial flutter). No language restrictions were set. Reference lists of included studies were carefully checked to identify additional studies.

### Inclusion and exclusion criteria

Eligible RCTs had to meet the following criteria: (i) enroll patients aged 18 years or older with a diagnosis of HFrEF [left ventricular ejection fraction (LVEF) ≤ 40%]; (ii) compare SGLT2i with placebo; and (iii) report at least one of the predefined outcomes of interest. RCTs focused on conditions other than HFrEF were excluded. Trial eligibility was assessed by two independent reviewers (D.S. and T.L.). Discrepancies between reviewers were resolved by consensus or, if necessary, by a third investigator (P.K.).

### Outcomes of interest

Primary outcomes of interest included the incidence of AF, AFL, and the composite of AF/AFL (new-onset and recurrent events of relevant arrhythmias) reported as serious adverse events according to Medical Dictionary for Drug Regulatory Activities (MedDRA). Prespecified subgroup analyses by SGLT2i agent used and follow-up duration were conducted to compare the SGLT2i treatment effect between different drug types and length of follow-up. Subgroup analysis by dosage was not performed because the included studies examined only the doses of 10 mg empagliflozin and 10 mg dapagliflozin.

### Data extraction and quality assessment

Two reviewers (D.S. and T.L.) independently performed data extraction using a standard form, including study characteristics, sample size, study design, follow-up duration, baseline characteristics of study population, interventions, comparisons, and outcomes of interest. Quality assessment of included RCTs was conducted using the Cochrane Risk of Bias Tool (performed by D.S. and T.L.). Discrepancies were resolved by consensus, or if necessary, by adjudication from a third investigator (P.K.).

### Statistical analysis

The collected raw data were used to calculate relative risks (RRs) and 95% confidence intervals (CIs), which were then pooled in meta-analyses. The pooled RRs and corresponding 95% CIs were used to present the incidence of AF, AFL, and the composite of AF/AFL. Heterogeneity was assessed using the Cochran Q statistic and Higgins and Thompson *I*^2^ before the meta-analysis. For the Q test, a *P* value < 0.1 was considered statistically significant. Additionally, *I*^2^ > 50% indicated at least moderate heterogeneity. If heterogeneity was present, as suggested by *P* value < 0.1 or *I*^2^ > 50%, the random-effects (RE) model was applied; otherwise, the fixed effects (FE) model was applied. All analyses were conducted using Review Manager (RevMan, version 5.4, The Cochrane Collaboration, 2020).

## Results

### Characteristics of eligible studies

Among the 2164 records identified, six RCTs [5, 6, 16–19] including 9467 patients with HFrEF (*N* = 4731 in the SGLT2i arms; *N* = 4736 in the placebo arms) matched the predefined inclusion criteria and were included in the final meta-analysis. The PRISMA flow diagram is presented in Supplementary material online, Appendix Fig. S1. The baseline characteristics of the included studies are summarized in Table 1. Except for two unpublished trials (DETERMINE-reduced; NCT03877237 [17] and EMPERIAL-reduced; NCT03448419 [18]), the other four trials [5, 6, 16, 19] have been published. Six RCTs [5, 6, 16–19] reported AF, and two RCTs [5, 6] reported AFL outcomes. No RCTs reported AF/AFL as a composite event. The mean age of the participants was 61.3–69 years, the proportion of males ranged from 73.3% to 76.6%, and the median follow-up duration ranged from 12 weeks to 18.2 months. Among the included studies, three trials [6, 16, 18] examined the effects of empagliflozin and three trials [5, 17, 19] the effects of dapagliflozin. Notably, only the doses of 10 mg empagliflozin and 10 mg dapagliflozin were examined.

**Table 1:** Baseline characteristics of eligible studies

**Study**	**Year**	**Study design (unique identifier)**	**Drug**	**Dose(s) analyzed**	**Inclusion criteria**	**Total number of trial participants**	**Type of control**	**Median follow-up duration**	**Age (years), mean**	**Male, n (%)**	**Patients with a history of T2DM, n (%)**	**Patients with a history of AF, n (%)**	**Patients with a history of AF/AFL**	**LVEF, mean, n (%)**
DAPA-HF	2019	RCT (NCT03036124)	Dapagliflozin	10 mg	HFrEF	4744	Placebo	18.2 months	66.3	76.6%	45.1%	38.3%	NA	31.1%
DETERMINE-reduced	2021	RCT (NCT03877237)	Dapagliflozin	10 mg	HFrEF	313	Placebo	16 weeks	67.8	74.4%	NA	NA	NA	NA
EMPERIAL-reduced	2020	RCT (NCT03448419)	Empagliflozin	10 mg	HFrEF	312	Placebo	12 weeks	69.0	74.4%	NA	NA	NA	NA
EMPEROR-Reduced	2020	RCT (NCT03057977)	Empagliflozin	10 mg	HFrEF	3730	Placebo	16 months	66.8	76.1%	49.7%	36.7%	NA	27.4%
SUGAR-DM-HF	2020	RCT (NCT03485092)	Empagliflozin	10 mg	HFrEF and T2DM or prediabetes	105	Placebo	36 weeks	68.7	73.3%	78.1% *	NA	NA	32.5%
DEFINE-HF	2019	RCT (NCT02653482)	Dapagliflozin	10 mg	HFrEF	263	Placebo	13 weeks	61.3	73.4%	63.1%	40.3%	NA	26.4%

^*^The rest of the patients (21.9%) had prediabetes

The results of the quality assessment are presented in Supplementary material online, Appendix Figs. S2 and S3. All the included RCTs were of high methodological quality. The participants included in the safety analysis set in some RCTs were fewer than those in the full analysis set, which yielded a 13-person difference between the baseline population (*N* = 9467) and the outcome evaluation population (*N* = 9454). Since the difference between the two sets was very small (0.13% of the total sample size), it was considered unlikely that it could have influenced the pooled results. Therefore, a low risk of bias was still considered.

### Incidence of atrial fibrillation

**Fig. 1:**
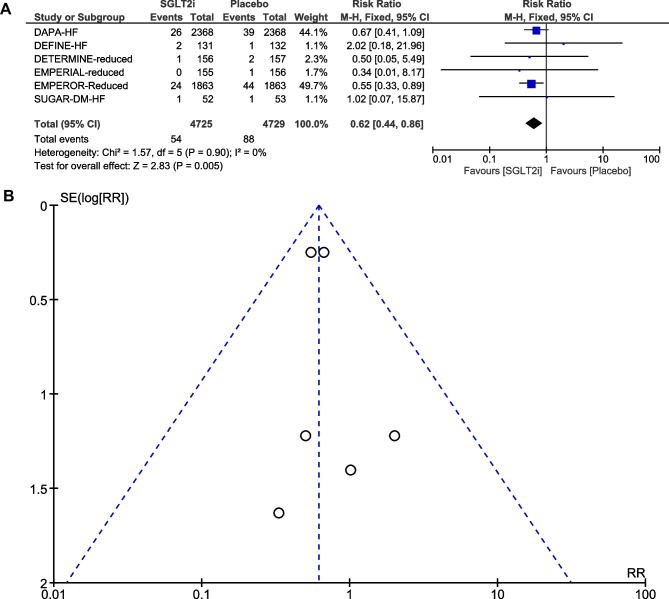
**A** Forest plot comparing the incidence of AF between SGLT2i and placebo. CI, confidence interval; AF, atrial fibrillation; SGLT2i, sodium-glucose cotransporter-2 inhibitors. **B** Funnel plot of meta-analysis for the incidence of AF. RR, relative risk; AF, atrial fibrillation; SE, standard error

**Fig. 2:**
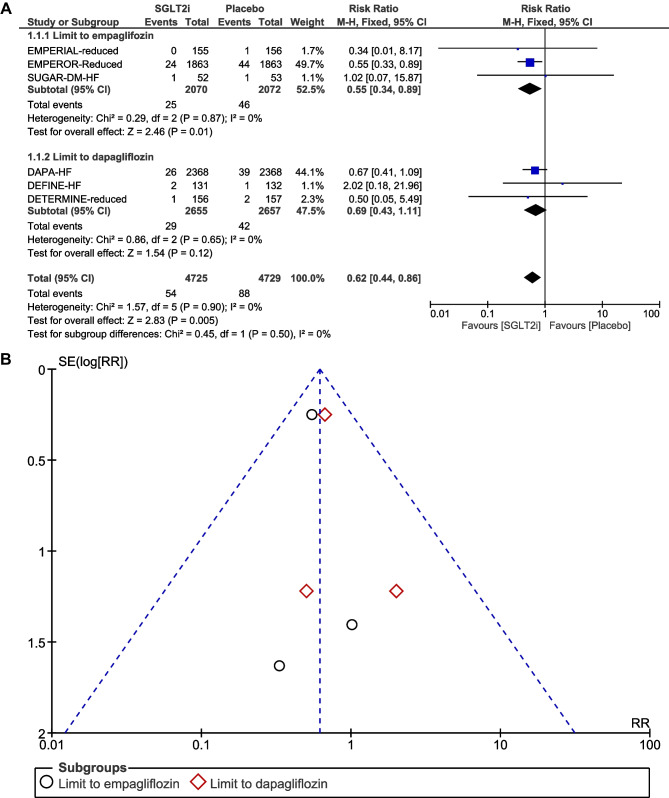
**A** Forest plot of subgroup analysis by SGLT2i agent used comparing the incidence of AF between SGLT2i and placebo. CI, confidence interval; AF, atrial fibrillation; SGLT2i, sodium-glucose cotransporter-2 inhibitors. **B** Funnel plot of subgroup analysis by SGLT2i agent used comparing the incidence of AF between SGLT2i and placebo. RR, relative risk; AF, atrial fibrillation; SE, standard error

**Fig. 3:**
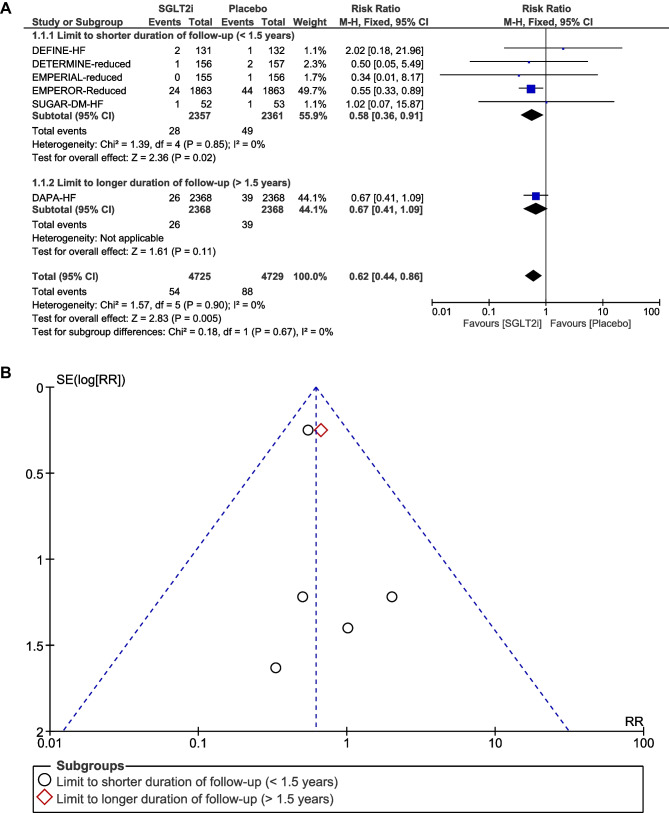
**A** Forest plot of subgroup analysis by follow-up duration comparing the incidence of AF between SGLT2i and placebo. CI, confidence interval; AF, atrial fibrillation; SGLT2i, sodium-glucose cotransporter-2 inhibitors. **B** Funnel plot of subgroup analysis by follow-up duration comparing the incidence of AF between SGLT2i and placebo. RR, relative risk; AF, atrial fibrillation; SE, standard error

AF was reported in six RCTs [5, 6, 16–19]. A total of 142 AF events were reported, of which 54 occurred in the SGLT2i group (*N* = 4725) and 88 in the placebo group (*N* = 4729). After pooling the six trials, treatment with SGLT2i was associated with a significant reduction in the risk of AF compared to placebo (RR, 0.62, 95% CI, 0.44–0.86; *P* = 0.005) (Fig. 1A). There was no significant heterogeneity across trials (*P* = 0.90; *I*^2^ = 0%). Visual inspection of the funnel plot suggested no apparent publication bias (Fig. 1B). In subgroup analysis by SGLT2i agent used, empagliflozin use resulted in a significant reduction in the risk of AF (RR, 0.55; 95% CI, 0.34–0.89; *P* = 0.01), whereas dapagliflozin use was not associated with a reduction in the risk of AF (RR, 0.69, 95% CI, 0.43–1.11; *P* = 0.12) (Fig. 2A and B). Given that there were five RCTs [6, 16–19] that had “shorter” duration of follow-up (< 1.5 years) and one RCT [5] that had “longer” duration of follow-up (> 1.5 years), subgroup analysis by follow-up duration was also conducted. When focusing the effects of follow-up duration on AF, “shorter” duration of follow-up (< 1.5 years; RR, 0.58; 95% CI, 0.36–0.91; *P* = 0.02) remained associated with a significant reduction in the risk of AF (Fig. 3A and B). In subgroup analysis by SGLT2i agent used, and by follow-up duration, the χ^2^ test for subgroup differences identified no significant subgroup effect (*P* = 0.50 and *P* = 0.67, respectively).

### Incidence of atrial flutter

AFL was reported in two RCTs [5, 6]. A total of 25 AFL events were reported, of which 11 in the SGLT2i group (*N* = 4231) and 14 in the placebo group (*N* = 4231). Since substantial heterogeneity was identified (*P* = 0.02; *I*^2^ = 83%), the two trials were pooled using the random-effects model. Overall, there was no significant difference between the SGLT2i arm and the placebo arm in the incidence of AFL (RR, 0.85; 95% CI, 0.09–7.92; *P* = 0.88) (Supplementary material online, Appendix Fig. S4). Visual inspection of the funnel plot revealed no asymmetry (Supplementary material online, Appendix Fig. S5).

### Incidence of the composite of atrial fibrillation and atrial flutter

**Fig. 4:**
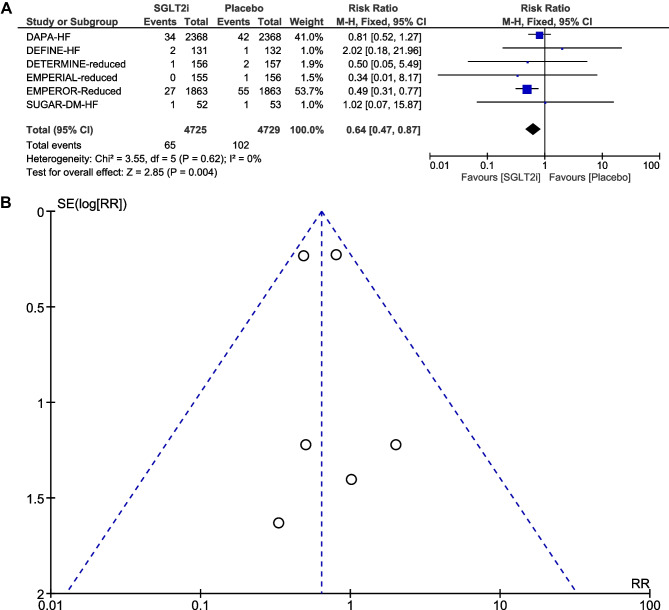
**A** Forest plot comparing the incidence of AF/AFL between SGLT2i and placebo. CI, confidence interval; AF, atrial fibrillation; AFL, atrial flutter; SGLT2i, sodium–glucose cotransporter-2 inhibitors. **B** Funnel plot of meta-analysis for the incidence of AF/AFL. RR, relative risk; AF, atrial fibrillation; AFL, atrial flutter; SE, standard error

**Fig. 5:**
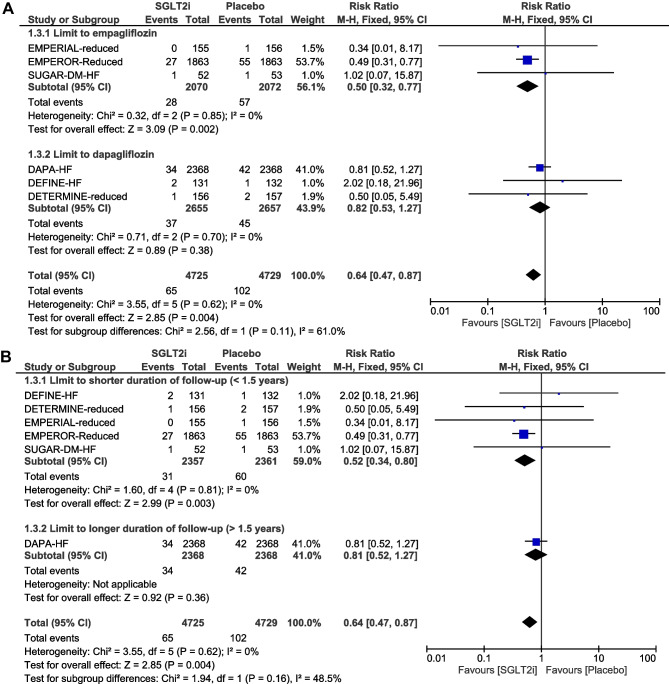
**A** Forest plot of subgroup analysis by SGLT2i agent used comparing the incidence of AF/AFL between SGLT2i and placebo. CI, confidence interval; AF, atrial fibrillation; AFL, atrial flutter; SGLT2i, sodium-glucose cotransporter-2 inhibitors. **B** Forest plot of subgroup analysis by follow-up duration comparing the incidence of AF/AFL between SGLT2i and placebo. CI, confidence interval; AF, atrial fibrillation; AFL, atrial flutter; SGLT2i, sodium-glucose cotransporter-2 inhibitors

When AF and AFL were combined as a composite endpoint, treatment with SGLT2i was associated with a significant reduction in the risk of AF/AFL compared to placebo (RR: 0.64, 95% CI: 0.47–0.87; *P* = 0.004) (Fig. 4A). There was no significant heterogeneity across trials (*P* = 0.62; *I*^2^ = 0%). Visual inspection of the funnel plot suggested no apparent publication bias (Fig. 4B). In subgroup analysis by SGLT2i agent used, empagliflozin use resulted in a significant reduction in the risk of AF/AFL (RR, 0.50; 95% CI, 0.32–0.77; *P* = 0.002), whereas dapagliflozin use was not associated with a significant reduction in the risk of AF/AFL (RR, 0.82; 95% CI, 0.53–1.27; *P* = 0.38) (Fig. 5 A and Supplementary material online, Appendix Fig. S6). Given that there were five RCTs [6, 16–19] that had “shorter” duration of follow-up (< 1.5 years) and one RCT [5] that had “longer” duration of follow-up (> 1.5 years), subgroup analysis by follow-up duration was also conducted. When focusing the effects of follow-up duration on the composite of AF/AFL, “shorter” duration of follow-up (< 1.5 years; RR, 0.52; 95% CI, 0.34–0.80; *P* = 0.003) was still associated with a significant reduction in the risk of AF/AFL (Fig. 5B and Supplementary material online, Appendix Fig. S7). In subgroup analysis by SGLT2i agent used, and by follow-up duration, the χ^2^ test for subgroup differences identified no significant subgroup effect (*P* = 0.11, and *P* = 0.16, respectively). Interestingly, there was substantial heterogeneity between the subgroups of empagliflozin and dapagliflozin (*I*^2^ = 61%) and moderate heterogeneity between the subgroups of shorter and longer duration of follow-up (*I*^2^ = 48.5%).

## Discussion

The present meta-analysis demonstrated that SGLT2i treatment was associated with a significant reduction in the risk of AF and AF/AFL in patients with HFrEF. In subgroup analysis by SGLT2i agent used, the use of empagliflozin, but not dapagliflozin, resulted in a significant reduction in the risk of AF and AF/AFL. In subgroup analysis by follow-up duration, a “shorter” duration (< 1.5 years) of treatment with SGLT2i remained associated with a significant reduction in the risk of AF and AF/AFL. As far as AFL is concerned, no significant association was observed.

AF, especially if new-onset or paroxysmal, confers a worse prognosis in patients with HFrEF [2, 20]. In fact, new-onset AF has been associated with a significantly increased risk of HF hospitalization and all-cause mortality, as well as cardiovascular mortality and stroke [2]. Similarly, paroxysmal AF has been shown to increase the risks of HF hospitalization and stroke, as well as pump-failure death [2]. Furthermore, the presence of AF in patients with HFrEF may reduce or abolish the benefits of beta-blockers and cardiac resynchronization therapy (CRT) and renders ivabradine ineffective [4]. In the same context, the Catheter Ablation Versus Standard Conventional Treatment in Patients With Left Ventricular Dysfunction and Atrial Fibrillation (CASTLE-AF) study indicated that maintenance of sinus rhythm in patients with HFrEF by catheter ablation of AF significantly reduces mortality [21]. Even though AFL is much less well studied, it is considered to have similar clinical significance and consequences to AF [3]. Therefore, prevention of AF and/or AFL is of particular importance in the setting of HFrEF.

The results of the present meta-analysis suggest that SGLT2i may have a favorable impact on the mechanistic pathways of AF and AF/AFL in patients with HFrEF. Although a significant association between SGLT2i and AFL was not observed, the number of AFL events was low, and this might have contributed to a wide confidence interval. Nonetheless, AFL is considered to have similar clinical significance and consequences to AF, and the combined analysis of AF/AFL showing a significant risk reduction might eliminate possible publication bias for AFL. In support of the results of this meta-analysis, SGLT2i have recently been shown to mitigate adverse cardiac remodeling and fibrosis [16, 22–24]. Furthermore, SGLT2i have been demonstrated to optimize loading conditions through their effect on natriuresis and diuresis [25]. Of note, SGLT2i have been shown to increase natriuresis without off-target electrolyte wasting, renal dysfunction, and neurohormonal activation [26]. In addition to the above, recent evidence suggests that SGLT2i might ameliorate HFrEF- and AF-related Na^+^ and Ca^2+^ handling abnormalities in the atrial cardiomyocyte level, which may prevent the triggering and maintenance of AF [27–29]. Even further, SGLT2i have been demonstrated to improve mitochondrial function and cardiac energy metabolism [30, 31]. Also, these agents ameliorate inflammation and oxidative stress through several mechanisms, including suppression of NLRP3 (NOD-, LRR-, and pyrin domain-containing protein 3) inflammasome and activation of 5′ AMP-activated protein kinase (AMPK) [30, 31]. Moreover, SGLT2i have been demonstrated to inhibit the sympathetic nervous system activity either directly or because of a reduction of renal afferent sympathetic activation [30, 32]. Nevertheless, SGLT2i have been shown to improve renal function which can indirectly improve cardiac function through a reduction in afferent sympathetic nervous system activation, attenuation of inflammation, and amelioration of oxidative stress [30]. Finally, SGLT2i have been shown to reduce epicardial fat mass, HbA1c, body weight, blood pressure, and uric acid levels, while they also increase erythropoietin levels and improve vascular function [30, 33].

### Limitations

This meta-analysis has several potential limitations. Firstly, AF and AFL were not the prespecified outcomes of the included trials; therefore, there might be ascertainment bias. Secondly, only AF or AFL events that were reported as serious adverse events according to MedDRA were included. Thirdly, the trials included were underpowered to study arrhythmia outcomes. Fourthly, the included trials examined only empagliflozin and dapagliflozin. Fifthly, most of the weight of the statistical analysis depended only on two studies, namely, EMPEROR-Reduced and DAPA-HF. Sixthly, most of the weight of the empagliflozin and dapagliflozin subgroups depended on the EMPEROR-Reduced and DAPA-HF trials, respectively, and therefore the results of subgroup analysis by agent used may have been influenced by the different populations included in them. Consequently, whether the use of empagliflozin results in a significant reduction in the risk of AF and AF/AFL, and dapagliflozin does not, needs to be further investigated. Seventhly, there were five RCTs that had shorter duration of follow-up (< 1.5 years), and only one RCT that had relatively longer duration of follow-up (> 1.5 years), restricting the significance of the results of subgroup analysis by follow-up duration. Finally, the included trials examined only the doses of 10 mg empagliflozin and 10 mg dapagliflozin, rendering subgroup analysis by dosage unfeasible. In this context, further research with adequately powered RCTs to study arrhythmia outcomes is needed.

## Conclusion

SGLT2i therapy was associated with a significant reduction in the risk of AF and AF/AFL in patients with HFrEF. These results reinforce the value of using SGLT2i in these patients. Further data with properly designed RCTs are needed to confirm the present findings, as well as to evaluate and elucidate the potential antiarrhythmic effects of SGLT2i in patients with HFrEF.

## Supplementary Information

Below is the link to the electronic supplementary material.

Supplementary file1 (DOCX 39 KB) Supplementary material online, Appendix Figure S1: Prisma flow diagram of study selection process

Supplementary file2 (PDF 238 KB) Supplementary material online, Appendix Figure S2: Results of the quality assessment (risk of bias graph)

Supplementary file3 (PDF 263 KB) Supplementary material online, Appendix Figure S3: Results of the quality assessment (risk of bias summary)

Supplementary file4 (PDF 257 KB) Supplementary material online, Appendix Figure S4: Forest plot comparing the incidence of AFL between SGLT2i and placebo. CI, confidence interval; AFL, atrial flutter; SGLT2i, sodium–glucose cotransporter-2 inhibitors

Supplementary file5 (PDF 28 KB) Supplementary material online, Appendix Figure S5: Funnel plot of meta-analysis for the incidence of AFL. RR, relative risk; AFL, atrial flutter; SE, standard error

Supplementary file6 (PDF 58 KB) Supplementary material online, Appendix Figure S6: Funnel plot of subgroup analysis by SGLT2i agent used comparing the incidence of AF/AFL between SGLT2i and placebo. RR, relative risk; AF, atrial fibrillation; AFL, atrial flutter; SE, standard error

Supplementary file7 (PDF 86 KB) Supplementary material online, Appendix Figure S7: Funnel plot of subgroup analysis by follow-up duration comparing the incidence of AF/AFL between SGLT2i and placebo. RR, relative risk; AF, atrial fibrillation; AFL, atrial flutter; SE, standard error

## Data Availability

Data are available from the corresponding author on reasonable request.
