# Supplementary material for: Association between sodium-glucose cotransporter-2 inhibitors and incident atrial fibrillation/atrial flutter in heart failure patients with reduced ejection fraction: a meta-analysis of randomized controlled trials
Source: Heart Fail Rev. 2022 Oct 25;28(4):925–36. doi: 10.1007/s10741-022-10281-3 (PMC10289933; doi:10.1007/s10741-022-10281-3)
Supplement: Supplementary file 1 — Supplementary file1 (DOCX 39 KB) Supplementary material online, Appendix Figure S1: Prisma flow diagram of study selection process [file 10741_2022_10281_MOESM1_ESM.docx]

## Identification

Records identified through ClinicalTrials.gov database searching
(n = 177)

Records identified through PubMed database searching
(n = 1,987)

## Screening

Records screened
(n = 2,164)

Duplicates removed
(n = 34)

Records after duplicates removed

(n = 2,130)

Records excluded after title and abstract screening
(n = 2,027)

Full-text articles assessed for eligibility
(n = 103)

## Eligibility

## Included

Full-text articles excluded, with reasons
(n = 97)

Studies included in the meta-analysis
(n = 6)
