# Supplementary figures and images for: Association between sodium-glucose cotransporter-2 inhibitors and incident atrial fibrillation/atrial flutter in heart failure patients with reduced ejection fraction: a meta-analysis of randomized controlled trials
Source: Heart Fail Rev. 2022 Oct 25;28(4):925–36. doi: 10.1007/s10741-022-10281-3 (PMC10289933; doi:10.1007/s10741-022-10281-3)

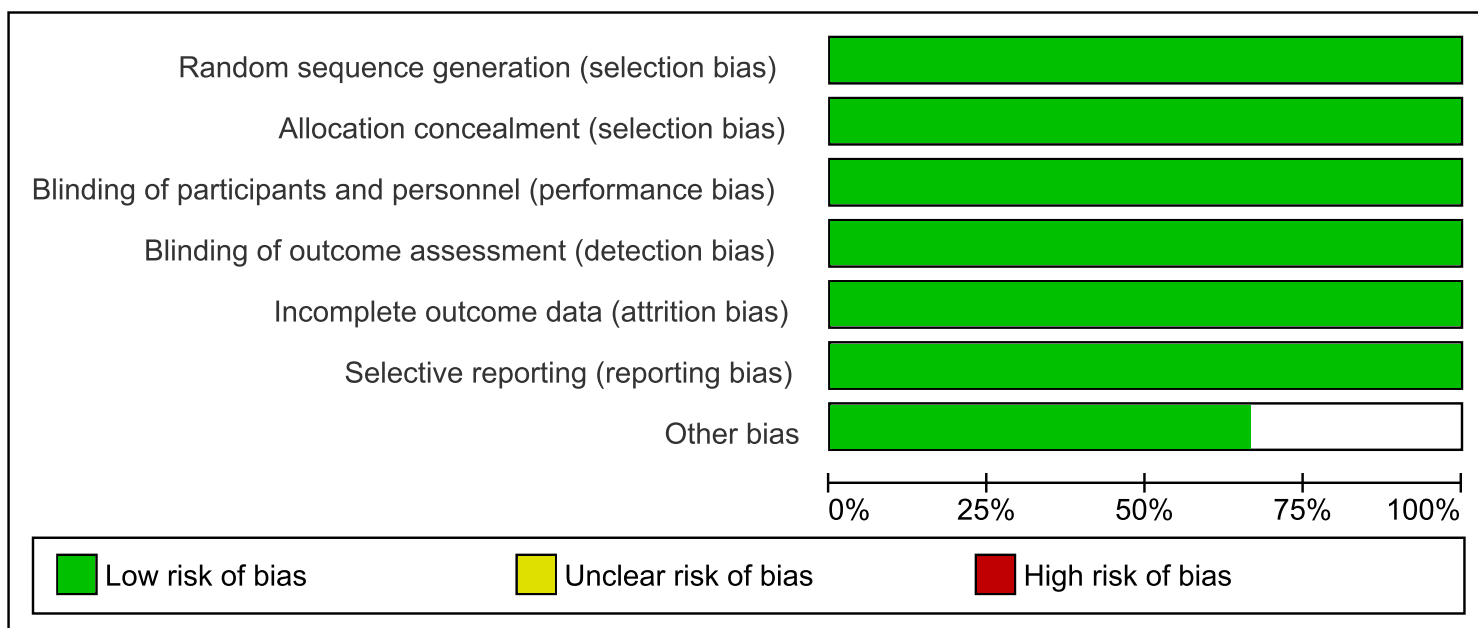

Supplement: Supplementary file 2 — Supplementary file2 (PDF 238 KB) Supplementary material online, Appendix Figure S2: Results of the quality assessment (risk of bias graph) [file 10741_2022_10281_MOESM2_ESM.pdf]

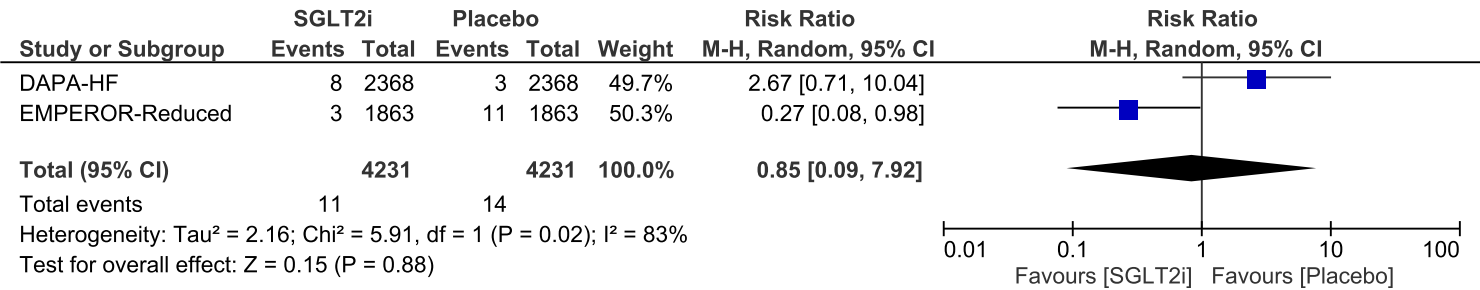

Supplement: Supplementary file 4 — Supplementary file4 (PDF 257 KB) Supplementary material online, Appendix Figure S4: Forest plot comparing the incidence of AFL between SGLT2i and placebo. CI, confidence interval; AFL, atrial flutter; SGLT2i, sodium–glucose cotransporter-2 inhibitors [file 10741_2022_10281_MOESM4_ESM.pdf]

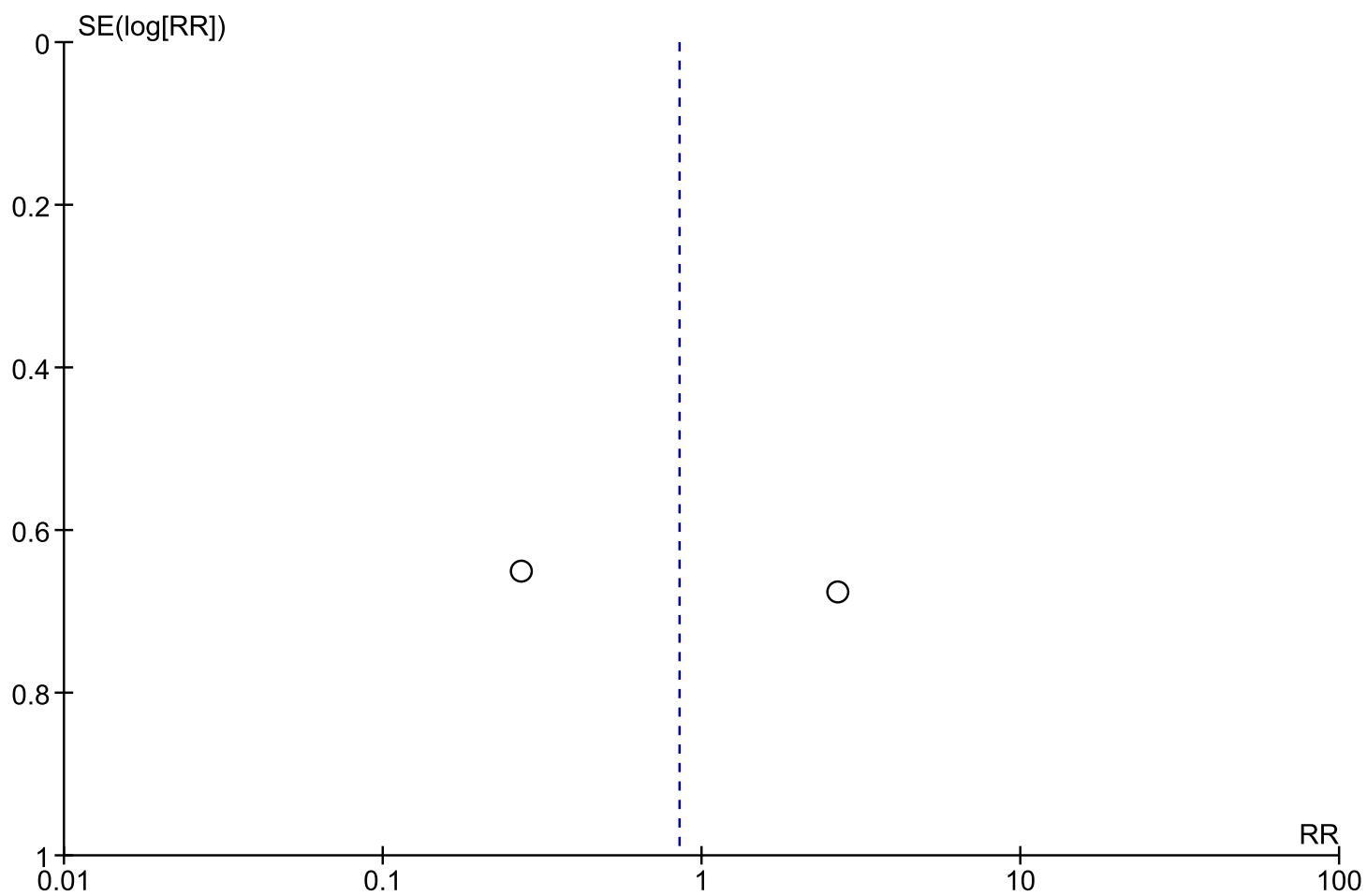

Supplement: Supplementary file 5 — Supplementary file5 (PDF 28 KB) Supplementary material online, Appendix Figure S5: Funnel plot of meta-analysis for the incidence of AFL. RR, relative risk; AFL, atrial flutter; SE, standard error [file 10741_2022_10281_MOESM5_ESM.pdf]

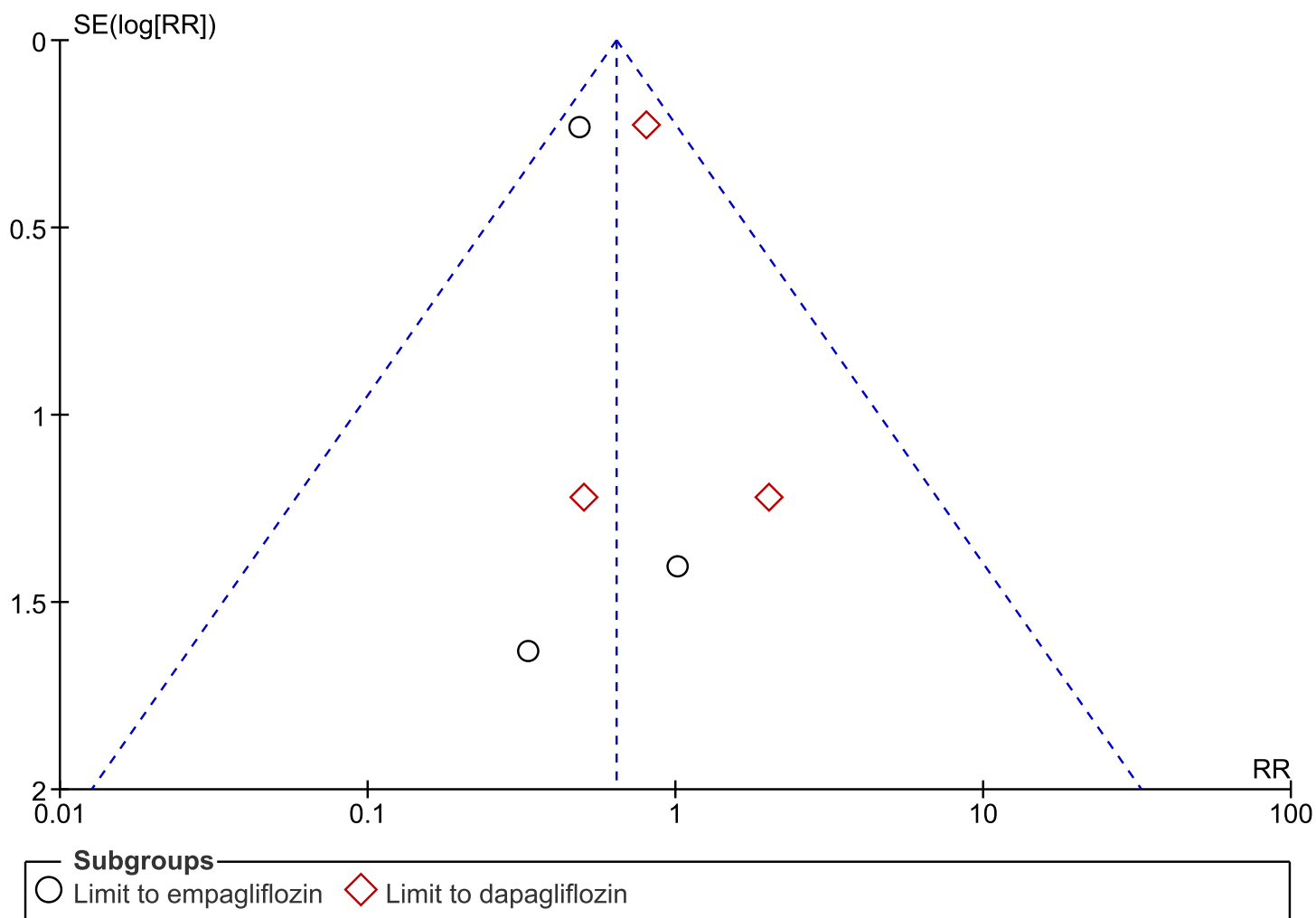

Supplement: Supplementary file 6 — Supplementary file6 (PDF 58 KB) Supplementary material online, Appendix Figure S6: Funnel plot of subgroup analysis by SGLT2i agent used comparing the incidence of AF/AFL between SGLT2i and placebo. RR, relative risk; AF, atrial fibrillation; AFL, atrial flutter; SE, standard error [file 10741_2022_10281_MOESM6_ESM.pdf]
