# Supplementary material for: Association between sodium-glucose cotransporter-2 inhibitors and incident atrial fibrillation/atrial flutter in heart failure patients with reduced ejection fraction: a meta-analysis of randomized controlled trials
Source: Heart Fail Rev. 2022 Oct 25;28(4):925–36. doi: 10.1007/s10741-022-10281-3 (PMC10289933; doi:10.1007/s10741-022-10281-3)
Supplement: Supplementary file 3 — Supplementary file3 (PDF 263 KB) Supplementary material online, Appendix Figure S3: Results of the quality assessment (risk of bias summary) [file 10741_2022_10281_MOESM3_ESM.pdf]

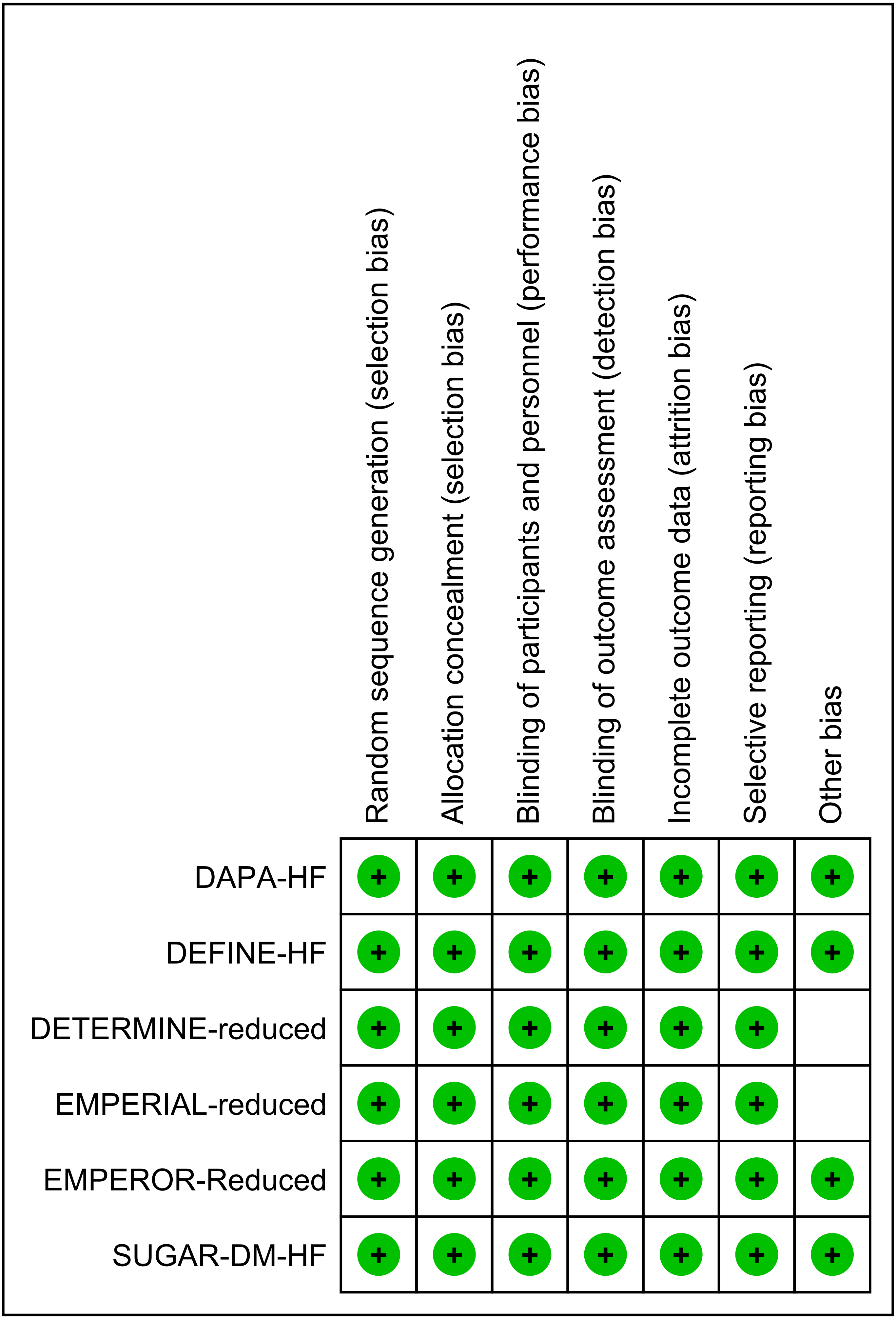
Random sequence generation (selection bias)
Allocation concealment (selection bias)
Blinding of participants and personnel (performance bias)
Blinding of outcome assessment (detection bias)
Incomplete outcome data (attrition bias)
Selective reporting (reporting bias)
Other bias
DAPA-HF
DEFINE-HF
DETERMINE-reduced
EMPERIAL-reduced
EMPEROR-Reduced
SUGAR-DM-HF
