# Supplementary material for: Association between sodium-glucose cotransporter-2 inhibitors and incident atrial fibrillation/atrial flutter in heart failure patients with reduced ejection fraction: a meta-analysis of randomized controlled trials
Source: Heart Fail Rev. 2022 Oct 25;28(4):925–36. doi: 10.1007/s10741-022-10281-3 (PMC10289933; doi:10.1007/s10741-022-10281-3)
Supplement: Supplementary file 7 — Supplementary file7 (PDF 86 KB) Supplementary material online, Appendix Figure S7: Funnel plot of subgroup analysis by follow-up duration comparing the incidence of AF/AFL between SGLT2i and placebo. RR, relative risk; AF, atrial fibrillation; AFL, atrial flutter; SE, standard error [file 10741_2022_10281_MOESM7_ESM.pdf]

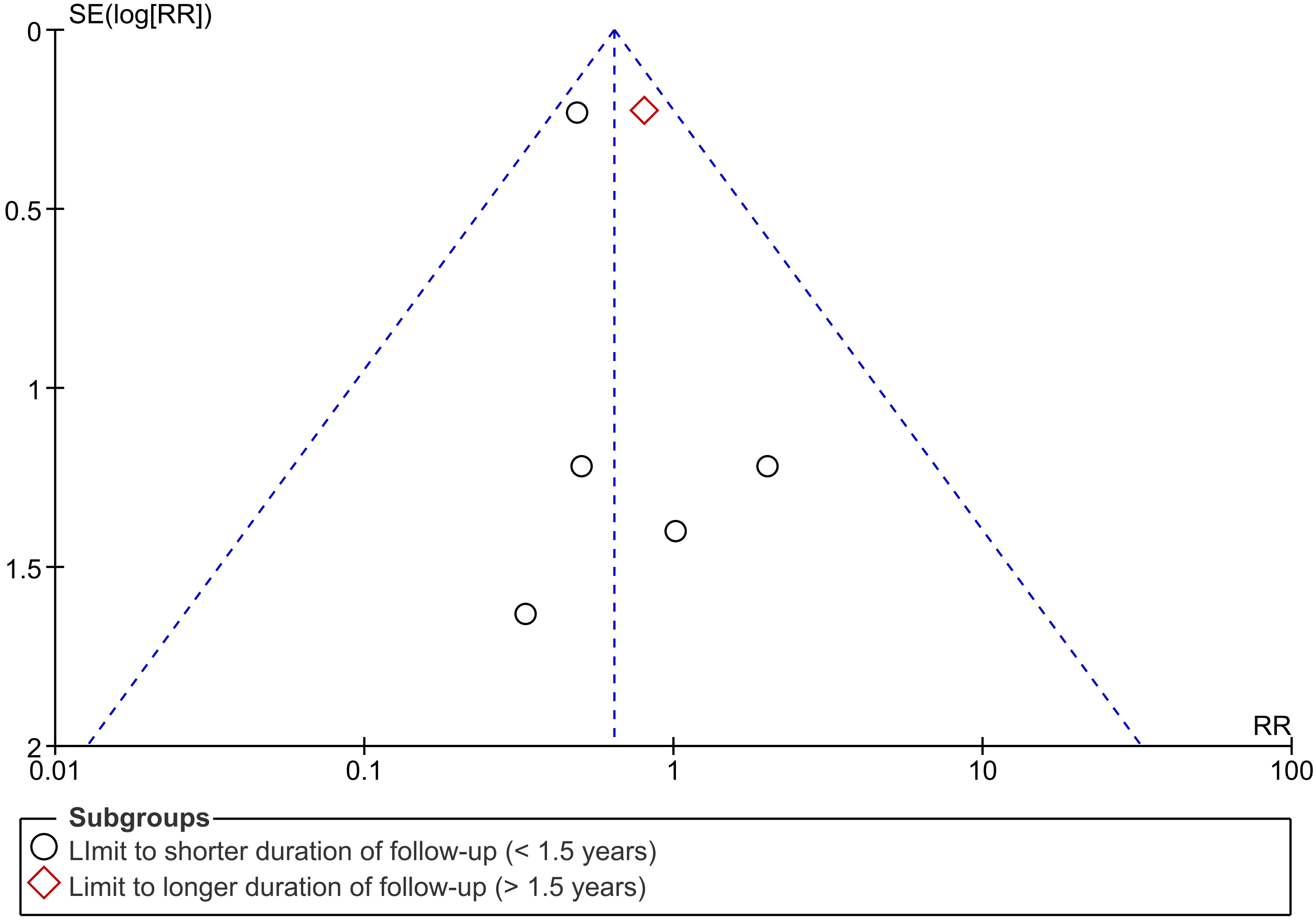
SE(log[RR])
0
0.5
1
1.5
2
RR
0.01
0.1
1
10
100
Subgroups
LImit to shorter duration of follow-up (< 1.5 years)
Limit to longer duration of follow-up (> 1.5 years)
